# Supplementary material for: Reversible molecular simulation for training classical and machine learning force fields
Source: Proc Natl Acad Sci U S A. Author manuscript; Available in PMC 2025 Jun 11. (PMC12146726; doi:10.1073/pnas.2426058122)
Supplement: Supplementary Materials [file EMS204898-supplement-Supplementary_Materials.pdf]

Reversible molecular simulation for training classical and machine learning force fields

Joe G Greener

Supplementary Methods and Data

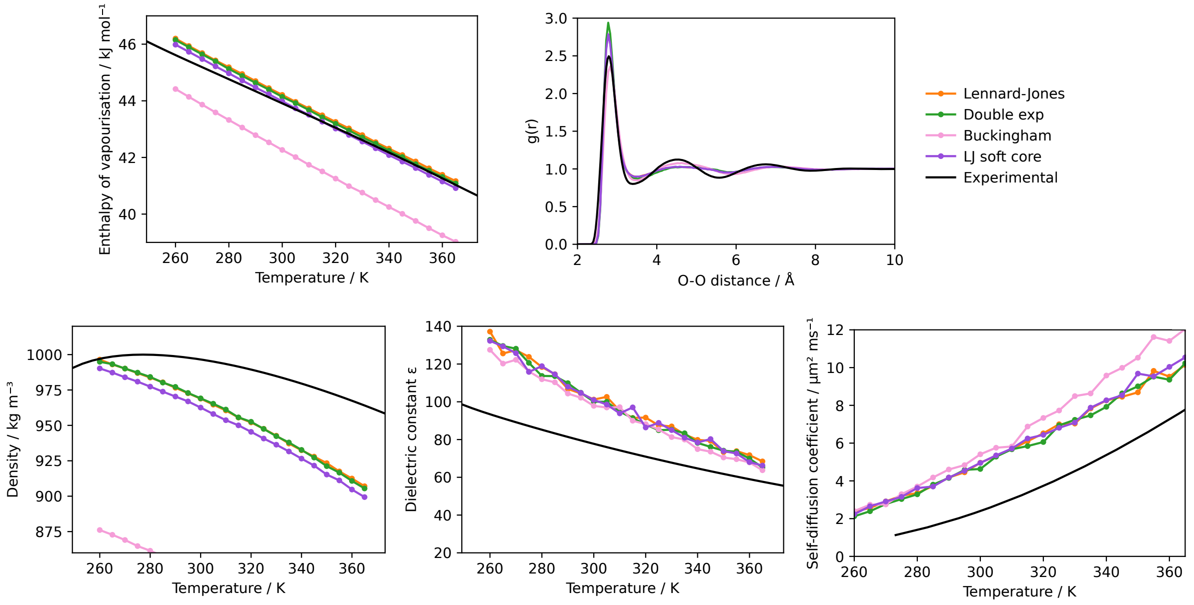

Figure S1 Validating oxygen diffusion models on condensed phase properties of bulk water with no oxygen molecules present. See Figure 3 for more details.

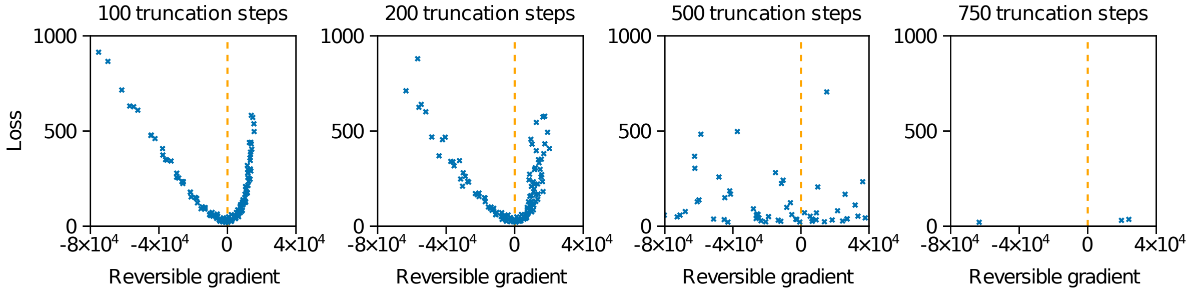

Figure S2 The effect of the number of steps after which gradients are truncated on gradient accuracy. A simulation of 1000 steps with a single loss snapshot was run with various parameters as described in Figure 1. The gradients were truncated after a given number of steps and the losses are shown plotted against the gradients. Some values are outside the range shown. 200 truncation steps were used throughout the results.

Reversible molecular simulation derivation

Consider a single simulation step of the Langevin middle integrator as shown in Equation 2. One operation at a time can be explicitly computed with constants represented by values of k and intermediate vectors represented by values of $\mathbf{w}$:

$$
\begin{aligned}
k_1 &= \frac{\Delta t}{2} \\
k_2 &= e^{-\gamma \Delta t} \\
k_3 &= \sqrt{1 - k_2^2} \\
\mathbf{f}_1 &= F(\mathbf{x}_1, \sigma_j) \\
\mathbf{a}_1^{'} &= \mathbf{f}_1 / \mathbf{m} \\
\mathbf{w}_1 &= \mathbf{a}_1^{'} \Delta t \\
\mathbf{w}_2 &= \mathbf{v}_{0+1/2} + \mathbf{w}_1 \\
\mathbf{w}_3 &= k_1 \mathbf{w}_2 \\
\mathbf{w}_4 &= \mathbf{x}_1 + \mathbf{w}_3 \\
\mathbf{w}_5 &= k_3 \mathbf{n}_1 \\
\mathbf{w}_6 &= k_2 \mathbf{w}_2 \\
\mathbf{v}_{1+1/2} &= \mathbf{w}_5 + \mathbf{w}_6 \\
\mathbf{w}_7 &= k_1 \mathbf{v}_{1+1/2} \\
\mathbf{x}_2 &= \mathbf{w}_4 + \mathbf{w}_7 \\
l &= L(\mathbf{x}_2, \mathbf{v}_{1+1/2}, \sigma_j)
\end{aligned}
$$

The multi-variable chain rule can then be used to compute $\frac{\mathrm{d}l}{\mathrm{d}\sigma_j}$:

$$
\begin{aligned}
\frac{\mathrm{d}l}{\mathrm{d}\sigma_j} &= \frac{\partial l}{\partial \sigma_j} + \frac{\mathrm{d}l}{\mathrm{d}\mathbf{f}_1}^{\top} \frac{\partial F(\mathbf{x}_1, \sigma_j)}{\partial \sigma_j} \\
\frac{\mathrm{d}l}{\mathrm{d}\mathbf{f}_1} &= \frac{\mathrm{d}l}{\mathrm{d}\mathbf{a}_1^{'}}^{\top} \frac{\mathrm{d}\mathbf{a}_1^{'}}{\mathrm{d}\mathbf{f}_1} = \frac{1}{\mathbf{m}} \frac{\mathrm{d}l}{\mathrm{d}\mathbf{w}_1}^{\top} \frac{\mathrm{d}\mathbf{w}_1}{\mathrm{d}\mathbf{a}_1^{'}} = \frac{\Delta t}{\mathbf{m}} \frac{\mathrm{d}l}{\mathrm{d}\mathbf{w}_2}^{\top} \frac{\mathrm{d}\mathbf{w}_2}{\mathrm{d}\mathbf{w}_1} = \frac{\Delta t}{\mathbf{m}} \left(\frac{\mathrm{d}l}{\mathrm{d}\mathbf{w}_3}^{\top} \frac{\mathrm{d}\mathbf{w}_3}{\mathrm{d}\mathbf{w}_2} + \frac{\mathrm{d}l}{\mathrm{d}\mathbf{w}_6}^{\top} \frac{\mathrm{d}\mathbf{w}_6}{\mathrm{d}\mathbf{w}_2} \right) \\
&= \frac{\Delta t}{\mathbf{m}} \left(\frac{\Delta t}{2} \frac{\mathrm{d}l}{\mathrm{d}\mathbf{w}_4} + e^{-\gamma \Delta t} \frac{\mathrm{d}l}{\mathrm{d}\mathbf{v}_{1+1/2}} \right) = \frac{\Delta t}{\mathbf{m}} \left(\frac{\Delta t}{2} \frac{\mathrm{d}l}{\mathrm{d}\mathbf{x}_2} + e^{-\gamma \Delta t} \frac{\mathrm{d}l}{\mathrm{d}\mathbf{w}_7}^{\top} \frac{\mathrm{d}\mathbf{w}_7}{\mathrm{d}\mathbf{v}_{1+1/2}} + e^{-\gamma \Delta t} \frac{\mathrm{d}l}{\mathrm{d}\mathbf{v}_{1+1/2}} \right) \\
&= \frac{\Delta t^2}{2\mathbf{m}} \left(1 + e^{-\gamma \Delta t} \right) \frac{\mathrm{d}l}{\mathrm{d}\mathbf{x}_2} + \frac{\Delta t}{\mathbf{m}} e^{-\gamma \Delta t} \frac{\mathrm{d}l}{\mathrm{d}\mathbf{v}_{1+1/2}}
\end{aligned}
$$

This is the first term in Equation 4. Following a similar process with assistance from Symbolics.jl [66] further terms, which quickly increase in complexity, can be derived. Examining the relationship between these terms manually leads to the relations in Equation 5.

Force gradients

The Lennard-Jones potential between two atoms is defined by potential energy V for interatomic distance r and atom pair parameters σ and ε. The magnitude of the force F and the gradients

required for reversible simulation are given by:

$$V(r,\sigma,\varepsilon) = 4\varepsilon\left[\left(\frac{\sigma}{r}\right)^{12} - \left(\frac{\sigma}{r}\right)^{6}\right]$$

$$F(r,\sigma,\varepsilon) = -\frac{\mathrm{d}V(r,\sigma,\varepsilon)}{\mathrm{d}r} = \frac{24\varepsilon}{r}\left[2\left(\frac{\sigma}{r}\right)^{12} - \left(\frac{\sigma}{r}\right)^{6}\right]$$

$$\frac{\mathrm{d}F(r,\sigma,\varepsilon)}{\mathrm{d}r} = -\frac{24\varepsilon}{r^2}\left[26\left(\frac{\sigma}{r}\right)^{12} - 7\left(\frac{\sigma}{r}\right)^{6}\right]$$

$$\frac{\mathrm{d}F(r,\sigma,\varepsilon)}{\mathrm{d}\sigma} = \frac{144\varepsilon}{r\sigma}\left[4\left(\frac{\sigma}{r}\right)^{12} - \left(\frac{\sigma}{r}\right)^{6}\right]$$

$$\frac{\mathrm{d}F(r,\sigma,\varepsilon)}{\mathrm{d}\varepsilon} = \frac{24}{r}\left[2\left(\frac{\sigma}{r}\right)^{12} - \left(\frac{\sigma}{r}\right)^{6}\right]$$

Significant computation can be reused when calculating these quantities. Note that when $r \gg \sigma$ the power 12 term will approach zero and $\frac{\mathrm{d}F}{\mathrm{d}\sigma}$ and $\frac{\mathrm{d}F}{\mathrm{d}\varepsilon}$ will have the same sign.

Comparison to ensemble reweighting

Consider for example the ForceBalance approach [9–11]. If l is a generic thermodynamic average property then:

$$\langle l \rangle = \sum_{i=1}^{M} l(\mathbf{x}_i,\sigma_j)p_i(\mathbf{x}_i,\sigma_j)$$

$$= \sum_{i=1}^{M} \frac{l(\mathbf{x}_i,\sigma_j)\exp\left(-\frac{E_i(\mathbf{x}_i,\sigma_j)}{k_B T}\right)}{Q}$$

$$Q = \sum_{k=1}^{M} \exp\left(-\frac{E_k(\mathbf{x}_k,\sigma_j)}{k_B T}\right)$$

where M is the number of microstates, p_i is the probability of state i, E_i is the potential energy of state i, Q is the partition function and the angle brackets represent the average over microstates. By differentiating this [9–11] we obtain:

$$\frac{\mathrm{d}\langle l \rangle}{\mathrm{d}\sigma_j} = \left\langle \frac{\partial l}{\partial \sigma_j} \right\rangle + \frac{1}{k_B T}\left(\left\langle l\frac{\mathrm{d}E}{\mathrm{d}\sigma_j}\right\rangle - \langle l \rangle \left\langle \frac{\mathrm{d}E}{\mathrm{d}\sigma_j}\right\rangle\right)$$

This can be compared to Equation 1. Finite differences can be used to calculate $\frac{\partial l}{\partial \sigma_j}$ and $\frac{\mathrm{d}E}{\mathrm{d}\sigma_j}$ [9], but AD provides a way to do this faster and with higher accuracy [6]. Typically, one or more simulations are run and the snapshots sampled are taken as representative of the microstates. This assumes sufficient sampling of low energy regions and requires enough time between snapshots to reduce correlation. The first term is the same as in Equation 1 and represents the direct dependence of l on the parameters. The second term represents how a change in the parameters affects the weighting of states in the ensemble. Reversible simulation does this by differentiating through a simulation, whereas the ensemble reweighting approach reweights the snapshots based on how the potential energy depends on the parameters. Ensemble reweighting therefore only consider snapshot states, whereas reversible simulation considers a number of steps prior to each snapshot state depending on gradient truncation. Since reordering states does not change the gradients arising from ensemble reweighting, observables that depend on multiple time points such as diffusion coefficients are not directly applicable to this scheme. DiffTRe extends the above approach by using thermodynamic perturbation theory to reuse states, allowing for more efficient training [6].

Comparison to the adjoint method

The adjoint method differentiates an ordinary differential equation (ODE) before discretising it [45, 38]. Consider a loss function L whose input is the result of an ODE solver acting on hidden state $\mathbf{z}$:

$$l = L(\mathbf{z}(t_1)) = L\left(\mathbf{z}(t_0) + \int_{t_0}^{t_1} f(\mathbf{z}(t), \sigma_j)dt\right)$$

The adjoint $\mathbf{a}(t)$ determines the gradient of the loss with respect to $\mathbf{z}(t)$:

$$\mathbf{a}(t) = \frac{\partial l}{\partial \mathbf{z}(t)}$$

It can then be shown [45] that:

$$\frac{\mathrm{d}\mathbf{a}(t)}{\mathrm{d}t} = -\mathbf{a}(t)^\top \frac{\partial f(\mathbf{z}(t), \sigma_j)}{\partial \mathbf{z}(t)}$$

$$\frac{\mathrm{d}l}{\mathrm{d}\sigma_j} = -\int_{t_1}^{t_0} \mathbf{a}(t)^\top \frac{\partial f(\mathbf{z}(t), \sigma_j)}{\partial \sigma_j} dt$$

The required integrals for solving $\mathbf{z}$, $\mathbf{a}$ and $\frac{\mathrm{d}l}{\mathrm{d}\sigma_j}$ can be computed in a single call to an ODE solver. This steps back through time starting from the final state, similar to reversible simulation. The two vector-Jacobian products above are similar to the two in Equation 5. However, reversible simulation discretises the differential equation before differentiating it [38]. This means that the gradients match those of the forward simulation to within numerical error. By contrast, the adjoint method solves a different equation to obtain the gradients, which can cause problems [48, 49]. It can be unclear how to best solve this adjoint equation. The forward simulation is stable for conventional MD cases, but this is not guaranteed for the adjoint equation [50], so it makes sense to use the gradients of the forward simulation if possible. There has also been work on second order neural ODEs [51].
